# Launching a salt substitute to reduce blood pressure at the population level: a cluster randomized stepped wedge trial in Peru

**DOI:** 10.1186/1745-6215-15-93

**Published:** 2014-03-25

**Authors:** Antonio Bernabe-Ortiz, Francisco Diez-Canseco, Robert H Gilman, María K Cárdenas, Katherine A Sacksteder, J Jaime Miranda

**Affiliations:** 1 CRONICAS Center of Excellence in Chronic Diseases, Universidad Peruana Cayetano Heredia, Av. Armendariz 497, Miraflores, Lima 18, Peru; 2 School of Public Health and Administration, Universidad Peruana Cayetano Heredia, Av. Honorio Delgado 430, Ingenieria, Lima 31, Peru; 3 Department of International Health, Johns Hopkins Bloomberg School of Public Health, Johns Hopkins University, 615 N Wolfe Street, Baltimore, MD, USA; 4 Área de Investigación y Desarrollo, Asociación Benéfica PRISMA, Carlos Gonzales 251, Maranga, Lima 32, Peru; 5 Department of Medicine, School of Medicine, Universidad Peruana Cayetano Heredia, Av. Honorio Delgado 430, Ingenieria, Lima 31, Peru

**Keywords:** Blood pressure, Hypertension, Operations research, Peru, Potassium chloride, Sodium-restricted diet, Trial

## Abstract

**Background:**

Controlling hypertension rates and maintaining normal blood pressure, particularly in resource-constrained settings, represent ongoing challenges of effective and affordable implementation in health care. One of the strategies being largely advocated to improve high blood pressure calls for salt reduction strategies. This study aims to estimate the impact of a population-level intervention based on sodium reduction and potassium increase – in practice, introducing a low-sodium, high-potassium salt substitute – on adult blood pressure levels.

**Methods/Design:**

The proposed implementation research study includes two components: Phase 1, an exploratory component, and Phase 2, an intervention component. The exploratory component involves a triangle taste test and a formative research study designed to gain an understanding of the best implementation methods. Phase 2 involves a pragmatic stepped wedge trial design where the intervention will be progressively implemented in several clusters starting the intervention randomly at different times. In addition, we will evaluate the implementation strategy using a cost-effectiveness analysis.

**Discussion:**

This is the first project in a Latin-American setting to implement a salt substitution intervention at the population level to tackle high blood pressure. Data generated and lessons learnt from this study will provide a strong platform to address potential interventions applicable to other similar low- and middle-income settings.

**Trial registration:**

This study is registered in ClinicalTrials.gov NCT01960972.

## Background

Hypertension is a silent condition and a major worldwide contributor to the growing pandemic of cardiovascular disease (CVD) and stroke, especially in low- and middle-income countries (LMIC) [1-3]. Poor control rates for hypertension and appropriate strategies to maintain normal blood pressure, particularly in resource-constrained settings, reflect the challenge of effective and affordable implementation in health care systems.

One of the policies being advocated by both international technical organizations and academic leaders is the implementation of salt reduction strategies [4,5]. This approach is applicable not only to LMIC but also established economies. The Institute of Medicine labeled hypertension as a ‘neglected disease’ and recommended salt-reduction strategies at the population level as the way forward [6,7].

A decade ago, the Prospective Studies Collaboration conducted a meta-analysis of 61 observational studies of blood pressure and vascular disease in adults and found that for each 2 mmHg decrease in systolic blood pressure, stroke mortality and cardiovascular mortality decreased by 10% and 7%, respectively, an effect that was observed in reductions of systolic blood pressure levels up to 115 mmHg [8]. This indicates that small changes in blood pressure at the population level could result in large public health gains. The main challenge, however, is how best to introduce and achieve these changes under real life conditions while acknowledging the potential impact of local contexts.

Sodium chloride has been the subject of intense scientific research aimed at understanding its impact on health, especially on blood pressure [9-14]. Although criticized because of a biased approach [15], the Committee on the Consequences of Sodium Reduction in Populations convened by the Institute of Medicine reported that existing studies that explore the impact of salt reduction strategies on health are less than optimal [6]. A recent systematic review assessing the effect of modest reduction in salt intake on blood pressure supports the need of reducing salt [10]. Two previous studies, one in Tibet [16] and another in rural China [17], have demonstrated the potential effect of salt substitution on blood pressure among individuals with diagnosed hypertension; however, to our knowledge, limited evidence regarding this effect is available at the population level.

The most recent WHO guidelines regarding sodium intake for adults and children [18] strongly recommend a reduction to less than 5 grams/day of salt among adults with or without hypertension. Yet, the successful implementation of this recommendation, especially in developing countries, might be difficult to achieve without taking into account the role of salt on palatability of foods associated with taste [19,20]. In Peru, no accurate data exists about the sources of salt consumption [21]. Based on our pilot studies, salt added during cooking, rather than processed foods, appears as the main source of salt intake. Monitoring salt intake in both developing and developed countries poses major practical challenges [22]. In the Peruvian context, salt reduction strategies do have a role and should be addressed through their own channels. Such an approach does not conflict with salt substitution interventions, since cooking practices are highly prevalent and thus amenable to achieve changes in patterns of overall salt intake.

This study protocol addresses both ongoing scientific and practical implementation challenges by estimating the impact of a population-level intervention based on reducing sodium and increasing potassium – in practice, introducing a low-sodium, high-potassium salt substitute – on adult blood pressure levels. The information gathered in this study will provide strong, locally relevant information that may be applicable to other similar settings in Latin America and elsewhere.

## Methods/Design

### Objectives

This study aims to bridge a gap in implementation research by reducing blood pressure levels at the population level through the introduction of a community-wide intervention with a low-sodium, high-potassium salt substitute in a resource-constrained setting. Accordingly, the specific objectives of this study protocol are: i) to assess patterns of predisposition towards incorporating a salt substitute into daily cooking among villagers, authorities, and other potential stakeholders, in order to inform the structure of the intervention in the local communities and ensure successful implementation; ii) to implement and assess the impact of an intervention using a salt substitute on blood pressure at the population level using a stepped wedge trial design; and iii) to determine, if successful, the cost of this implementation and the incremental cost-effectiveness ratio of our intervention.

### Study design

The proposed implementation research study includes an exploratory component and a field intervention component. The intervention involves a pragmatic stepped wedge trial design, such that we will progressively implement our intervention at random in several clusters [23] following an introductory period oriented to gaining an understanding of the best implementation methods. The study will include both qualitative and quantitative methodologies for collecting baseline information, and monitoring the impact of the intervention during rollout and at its conclusion.

### Setting and time frame

This study involves inhabitants from Tumbes, a department located in the north coastal region of Peru on the border with Ecuador. The semi-urban area of Tumbes consists of more than 100 villages of varying size with an approximate total population of 80,000. It is comprised of a large ‘mestizo’ – mixed of European and Amerindian ancestry – population, and the traditional agricultural and fishing landscape has become intermixed with rapidly growing urban sections. Illiteracy rate is around 10%, and 50% of the inhabitants have no health insurance. Activities related to this protocol began in 2012 and are expected to continue through 2017.

### Participant recruitment and selection criteria

Participants from six randomly-selected villages will be involved in the different phases of the study. Potentially eligible subjects will be identified from the most updated census available. Males or females aged 18 years and over from the randomly selected villages, capable of understanding study procedures, capable of providing informed consent, and full-time residents in the area are eligible. Only those participants with mental illness that impair their consent will be excluded. In the case of the stepped wedge trial, self-reported history of chronic kidney disease and heart disease will be considered as additional exclusion criteria.

### Procedures

To accomplish the proposed objectives, this protocol has two phases:

#### *Phase 1: Exploratory phase*

This component includes a triangle taste test and a formative qualitative research study that will generate the elements to create a social marketing campaign before the intervention’s implementation.

##### 

**Triangle taste test** This test is a discriminative form of a sensory analysis that may indicate whether or not a detectable difference in taste exists between two samples [24]. Common salt, 100% sodium chloride, will be compared to salt substitute samples containing different potassium chloride concentrations: 25%, 33%, and 50%, with the consequent reduction in sodium. This experiment will allow the assessment of acceptability of taste of the salt substitutes compared to common salt. Participants will be exposed to three coded food samples, two identical and one different (odd). Subjects will taste each sample in a random order, to avoid positional bias, and will be instructed to identify the odd sample [25].

##### 

**Formative research** This component will combine focus groups and in-depth interview research techniques with a wide range of participants from the community including inhabitants, commercial traders, and stakeholders such as staff from the health care system as well as local authorities. In this way, we will guarantee a diversity of points of view regarding ordinary salt, high blood pressure, and CVD to create an appropriate marketing campaign to increase the likelihood of the acceptance of the salt substitute. The focus groups will include inhabitants from different villages, while in-depth interviews will be conducted with individuals with hypertension and stakeholders. The stakeholders include, but are not limited to, community health leaders and health promoters, local authorities (village representatives, health authorities, etc.), health workers (physicians, nurses, and health technicians), and commercial traders (restaurant owners, street vendors, community kitchen members, bakers, shopkeepers, etc.).

##### 

**Social marketing campaign** The goal of this campaign is to introduce participants to the salt substitute prior to and during the intervention in order to enhance its acceptance. Thus, villagers and stakeholders will be actively invited to develop and later be exposed to social marketing techniques designed to encourage the salt substitute consumption.

#### *Phase 2: Intervention and implementation activities*

##### 

**Baseline data collection** This information will be collected immediately before implementing activities in all participant villages. We plan to collect baseline information of participants at the individual- and family-level. We will use a modified version of the WHO STEP approach questionnaire for surveillance of non-communicable diseases [26]. Questions related to salt intake have also been included in these instruments. Ideally, salt intake should be assessed in urine and this is a limitation of our pragmatic design. We will indirectly evaluate salt intake consumption patterns by weighing salt containers in households. In addition, this baseline will include a clinical assessment to measure blood pressure in triplicate using standardized procedures with automated validated devices, and weight and height using standardized techniques and procedures. Costs-related data will also be collected at baseline in order to inform cost-effectiveness analysis at a later stage. A summary of the data collected at baseline is shown in Table 1.

**Table 1:** Sections and topics of the questionnaire in the study

**Section**	**Components**
Demographic assessment form:	- Place and date
	- Consent process
	- Contact information
Sociodemographic information form:	- Demographic information
	- Health coverage
Household information form:	- Family characteristics
	- Expenditures
	- Change attitudes
	- History of blood pressure measurements
Knowledge about salt and high blood pressure:	- Knowledge about high blood pressure
	- Salt consumption
	- Household information: assets
Lifestyles assessment form:	- Smoking
	- Alcohol consumption
	- Food consumption
	- Physical activity
	- Lack of activity
Mental health assessment form:	- Depressive symptoms
	- Quality of life
	- Stress
	- Sleep patterns
Cardiovascular assessment form:	- Cardiovascular medication
	- Personal and familiar history
	- Memory
	- Stroke
Clinical measurements form:	- Height
	- Weight
	- Waist and hip circumference
	- Blood pressure
Costing data form:	- Patient costs
	- Program costs

##### 

**Intervention and implementation activities** Salt replacement will be progressively implemented over six months in each village. The intervention will contemplate interactions with families as well as bakeries, community kitchens, food vendors including street vendors, and restaurants. Ideally, replacement will require a complete exchange of ordinary salt. The assessments of salt consumption will be carried out using questionnaires and weighing of salt containers at randomly selected households over time, and also by evaluating supply chain management indicators such as rate of delivery of the salt substitute to each family or food vendors.

Because of logistical constraints, especially around introducing and sustaining an adequate social marketing campaign together with the salt substitute delivery, the intervention can only be implemented in stages. Thus, the stepped wedge trial is an optimal design to ensure both moral and social acceptability. As described by Brown [23], “*in a stepped wedge design, an intervention is rolled-out sequentially to participants (either as individuals or clusters of individuals) over a number of time periods. The order in which the different individuals or clusters receive the intervention is determined at random and, by the end of the random allocation, all individuals or groups will have received the intervention. Stepped wedge designs incorporate data collection at each point where a new group (step) receives the intervention*”. In Table 2, villages receiving the intervention are indicated with the number 1, and those villages not receiving the intervention are indicated with a 0.

**Table 2:** Stepped wedge design for the proposed study

**Clusters**	**Time period**						
	**1**	**2**	**3**	**4**	**5**	**6**	**7**
Village 1	0	1	1	1	1	1	1
Village 2	0	0	1	1	1	1	1
Village 3	0	0	0	1	1	1	1
Village 4	0	0	0	0	1	1	1
Village 5	0	0	0	0	0	1	1
Village 6	0	0	0	0	0	0	1

##### 

**Randomization** Before the beginning of the implementation process, clusters will be randomly allocated a time when they are going to receive the intervention. According to the CONSORT guidelines extended to cluster randomized trials, the statistician will randomize the cluster using computer generated random numbers [27]. Only the research team will be aware of the allocation of all of the clusters. In order to keep allocation concealment, village inhabitants will be informed of the allocation time at the moment of the implementation in their cluster.

Due to the nature of the intervention, it will be not possible to blind inhabitants to the intervention. However, outcomes will be assessed by a different fieldwork team blinded to the intervention.

##### 

**Periodical assessments** These will include data collection regarding costs and clinical measurements. Every five to six months, once a new village commences on the intervention, a new evaluation will be performed in all participant villages, both intervention and control. Clinical measurements will include systolic and diastolic blood pressure, height, and weight. These periodic assessments will be performed at each household to guarantee contact with each family member enrolled in this study.

##### 

**Cost-effectiveness analysis** For this component, we will be interested in total cost of the implementation, including costs of the substitute itself and other associated costs due to transportation, delivery, and consumption frequency. We will also evaluate costs associated with the marketing and engagement campaign as well as capital costs. Information will be collected at the baseline but also in each of the periodic assessments. Thus, an incremental cost-effectiveness analysis as well as the additional cost to avoid an increase of 1 mmHg at the population level will be performed, comparing to the cost of using ordinary salt. This involves the estimation of the discrepancy in costs between the two contexts, ordinary salt and salt substitute consumption, and the difference of mmHg increase in each case.

### Sample size

#### *Triangle taste test*

Given that three coded food samples will be presented to each participant, two similar and one odd, there is a probability of 33% of randomly selecting the appropriate sample. However, if a participant can differentiate between the two types of samples, since two are similar, the actual probability is 50%. Assuming a significance level of 5% and a power of 90%, approximately 146 participants will be required, as described previously elsewhere [25].

##### 

**Formative research** For this component, approximately 228 participants are needed to have enough opinions in the focus groups (n = 168) and in-depth interviews (n = 60). Focus groups will include healthy and hypertensive participants from different villages stratified by sex and age (20 to 44 and 45 to 65 years old). Separately, in-depth interviews will be conducted among hypertensive individuals as well as community or local stakeholders. We expect to enroll approximately 10 participants from each village for the interviews. Of these, 5 will be individuals with hypertension, whereas the other 5 will be stakeholders, e.g., community health leaders and authorities, commercial traders, health workers, and others.

#### *Stepped wedge trial*

Calculations were derived using preliminary data from the baseline of the CRONICAS cohort study in Tumbes [28] and the PERU MIGRANT study estimates [29]. Power for the stepped wedge design was computed for a continuous endpoint [30], where X is a *N x T* matrix showing the treatment pattern, i.e., X_ij_ = 1 if cluster ‘i’ received the intervention at time ‘j’ and 0 otherwise. We assumed a significance level of 5%, a standard deviation of blood pressure within sites of 20 mmHg (σ), the number of clusters (N) as 6, the number of time periods (T) as 6 (excluding baseline assessment), the average number of subjects assessed per cluster and time period as 300, and τ, an approximation to the coefficient of variation, as 0.20. Based on those assumptions, we calculated a power over 90% to find a difference (θ) of 3 mmHg in blood pressure levels between the intervention and control groups. This magnitude of difference is within the expected range that provides major public health gains in the long-term, in particular in reduction of stroke [8]. Typically, the coefficient of variation ranges between 0.15 and 0.40, but when this value is unknown, as in this study, sensitivity of the sample size within this range needs to be verified [31]. In this protocol, power calculations using both extremes of coefficient of variation yields a power greater than 90%.

### Statistical analysis

#### *Triangle taste test*

We will be able to determine whether the total number of correct responses for the total number of participants is statistically significant based on the critical number of correct responses in a triangle taste test. Thus, there is a one out of three chance that the correct (odd) sample will be picked just by chance (guessing). We will then compute a z-statistic based on the following formula:

$$z=\frac{\left(k-\left(n/3\right)\right)}{\sqrt{2n/9}}$$

In this formula, ‘n’ is the total number of subjects assessed and ‘k’ is the number of correct responses [32]. Based on these results, we will reject the hypothesis of ‘no difference’ between samples if the number of correct responses is significantly greater than the calculated value (z).

##### 

**Qualitative data** Information from focus group sessions and in-depth interviews will be recorded and transcribed. All the collected information will be segmented, entered, coded, and analyzed using Atlas.Ti to identify a list of relevant themes and thus, identify key concepts to create our social marketing campaign. Coding will be carried out using the grounded theory approach to data analysis, where the data emerges from the participants, not the researchers [33,34]. The analysis will compare the information obtained from different subgroups of participants in order to identify and describe the similarities and divergences between men and women, patients from different age groups, and health workers and stakeholders. The analysis will also include notes from the field, i.e., fieldwork diary and minutes of the researchers’ meetings.

#### *Stepped wedge trial design*

Descriptive and exploratory statistics using tabulations and graphical methods will be derived. This process will allow us to verify the data entry process as well to detect atypical values. Following careful checking, we will describe variables of interest, especially our numeric outcome, using appropriate central tendency measures (mean, median, etc.) and dispersion measures (standard deviation, inter-quartile range, etc.). Appropriate longitudinal and panel data analysis techniques will be used for the assessment of blood pressure levels from baseline to the end of the study. Since cluster sizes may vary, an efficient analysis at the cluster mean level requires weights that depend on unknown parameters. Thus, an analysis at the individual level using generalized estimating equations (GEE) or generalized linear mixed models are preferable. Of these, GEE can flexibly handle normal or non-normal endpoints and tends to be more robust to misspecification of the variance structure since ‘sandwich’ type variance estimates are used [35]. In the same sense, GEE uses within-cluster and between-cluster information to estimate the treatment effect. This approach is necessary to avoid confounding the treatment effect with changes over time [36].

##### 

**Economic evaluation** Cost effectiveness analysis can be used as a tool for measuring costs and health gains of interventions, which in turn can help in decision-making and resource allocation. At the first stage, we will develop a cost analysis associated with the patient costs and the program implementation. Patient costs involve costs of illness data for treating high blood pressure or its complications, i.e., cardiovascular drugs, medical visits, hospitalization, laboratory tests, special diets, physical therapies, blood pressure caregiving, and monitors and other devices needed. Program costs will include marketing campaign, training, administration, production, and delivery of the salt substitute, among other activities. Our effect indicators will be measured by different health outcomes: blood pressure level (mmHg), number of cases averted (CVDs and stroke), and disability-adjusted life years averted. An incremental cost effectiveness ratio will show us the additional cost of a unit of health gained. Our cost-effectiveness analysis methodology will follow the guidelines established by WHO-CHOICE [37] and recommendations given by the Disease Control Priorities Project [38].

### Ethical issues

This project was reviewed and approved by Institutional Review Boards at Universidad Peruana Cayetano Heredia (Peru) and Johns Hopkins University (USA). Informed consent for participation will be obtained from all human subjects prior to subject participation and confidentiality of subjects will be protected.

All study participants will be linked to a unique identification code. Data will be recorded both in paper form and electronically. The electronic information will be stored, backed up, and secured by password protection. Paper forms will be stored in secure locked cabinets. All personal information, including participant’s names, addresses, and dates of birth, will be stored in a file and password protected as well. Only study investigators will have access to confidential information.

## Discussion

Developing effective pragmatic preventive strategies for chronic non-communicable diseases in resource-constrained settings is a challenge that needs to be addressed. In addition, there has been little research coverage and discussion of non-clinical, population-based approaches as an avenue to tackle CVDs in developing settings [39]. Therefore, our understanding of non-pharmacological intervention strategies for non-communicable diseases, such as hypertension, in resource-poor settings is limited, if not absent.

The complex context of LMIC can provide a variety of scenarios that could identify new areas for innovation, relevant at both local and international levels. For example, there are both challenges and opportunities related to salt substitution in Peru. A previous successful public health measure involved promoting iodine-supplemented salt as a vehicle to prevent iodine deficit disorders, such as goiter [40], and in commercial spheres, the slogan “*Consuma salud… consuma sal*”, or “Buy health… buy salt”, is being promoted. This is a challenge for our intervention because salt has been marketed as a positive health measure, and we now propose to promote the replacement of regular salt with a substitute. Changes associated with growth and urbanization also poses an additional challenge for implementation research. However, these circumstances also provide an opportunity, because the success of this measure demonstrates the successful collaboration of government and industry in Peru. This underscores the importance and relevance of understanding the local context, and we can utilize this knowledge taking advantage of this past successful partnership to explore the introduction of a salt substitute to be tested at a wide community intervention. Thus, the data gathered in this study will provide a strong platform to address potential interventions that are locally relevant and that could be applicable to other settings in Latin America and, eventually, to settings in other LMIC countries.

Previous reports demonstrated that salt reduction may play an important role in reducing blood pressure levels among hypertensive and normotensive people [13,41,42], and may reduce cardiovascular disease [43]. Similarly, the increase of dietary potassium can reduce mean systolic and diastolic blood pressure levels [44,45], and could contribute to the prevention of hypertension, especially in populations with elevated blood pressure [13,46]. However, as pointed out by the Committee on the Consequences of Sodium Reduction in Populations [6], most of the evidence on clinical outcomes came from observational prospective cohort studies. Moreover, data examining the effect of dietary sodium in combination with other electrolytes, particularly potassium, on health outcomes, is needed [6]. Thus, the results of this study are of potential interest as an approach to gradually reduce sodium intake in resource-constrained settings.

In most developed countries, a reduction in salt intake can be achieved by a gradual and sustained reduction in the amount of salt added to food by the food industry. In other countries, especially LMIC, where most of the salt consumed comes from salt added during cooking or from sauces, a different public health campaign would be required to encourage consumers to use less salt [47].

The study described here may be the first pragmatic intervention in a Latin-American country to implement a salt substitute at the population level, and it could yield a significant impact on public health. Replacing ordinary salt with a substitute containing low-sodium and high-potassium significantly reduced blood pressure levels among hypertensive participants of a randomized controlled trial conducted in a clinical setting in Tibet [16], as well as hypertensive and normotensive participants in rural areas of China [17,48]. In consequence, a salt substitute may be an effective adjuvant treatment for patients with hypertension and effective in preventing hypertension in normotensive individuals. Nevertheless, population-based approaches are needed to guarantee appropriate scaling-up in other contexts.

Strategies for salt intake reduction have been addressed as potentially very cost-effective measures [49]. In the long-term, a population-based approach, such as reducing salt intake, would have an effect in the entire population. Therefore, including larger population groups as beneficiaries of preventative interventions, and not only high-risk individuals, might render such approaches attractive because it could be more cost-effective. In the same vein, scaling up this intervention at the population level might offer a very simple, low-cost lifestyle approach to blood pressure reduction and control in resource-constrained settings [39].

The development and evaluation of a strategy for implementing the salt substitute at the community level, with participation of multiple stakeholders, will produce strong evidence to aid policy makers and public health specialists in the implementation of affordable prevention strategies at a LMIC level [50]. Our results will include a cost-effectiveness analysis component, which will provide more arguments for the policy debate. In planning for scalability, we are involving key decision makers at the central government level. We have also initiated communications with Peru’s Parliament representatives in order to make them aware, early in the process of research, of the potential public gains of these types of interventions. Closer to the health sector, we have enabled the interaction of the Peruvian Society for Nutrition as well as the Peruvian National Institute of Health. The latter is already tasked with ongoing monitoring of the salt supplementation with iodine, and will be a key foundation partner to explore potential links and discussions with industry.

## Trial status

Both phases of the study protocol have been reviewed and granted approval by the Institutional Review Boards at Universidad Peruana Cayetano Heredia and Johns Hopkins University. This study has been registered as a clinical trial (NCT01960972). As of this manuscript submission, we have completed Phase 1 of the project, and we plan to start Phase 2, enrollment into the stepped wedge trial, in March 2014.

## Abbreviations

CVD: Cardiovascular diseases; GEE: Generalized estimating equations; LMIC: Low- and middle income countries.

## Competing interests

The authors declare that no competing interests exist.

## Authors’ contributions

ABO, KAS, and JJM drafted the first version of the manuscript. ABO, FDC, MKC, and JJM designed the sampling procedures. ABO, FDC, RHG, KAS, and JJM conceived and designed the overall study. MKC designed the strategy for the cost-effectiveness analysis. All of the authors contributed to the revising of the manuscript for important content and gave their final approval of the version submitted for publication.
